# Maternal heterozygous mutation in *CHEK1* leads to mitotic arrest in human zygotes

**DOI:** 10.1007/s13238-021-00844-9

**Published:** 2021-05-04

**Authors:** Beili Chen, Jianying Guo, Ting Wang, Qianhui Lee, Jia Ming, Fangfang Ding, Haitao Li, Zhiguo Zhang, Lin Li, Yunxia Cao, Jie Na

**Affiliations:** 1 Department of Obstetrics and Gynecology, Reproductive Medicine Center, the First Affiliated Hospital of Anhui Medical University, Hefei, 230032 China; 2 School of Medicine, Tsinghua University, Beijing, 100084 China; 3 Department of Chemistry, Tsinghua University, Beijing, 100084 China; 4 Central Laboratory, Beijing Obstetrics and Gynecology Hospital, Capital Medical University, Beijing, 100026 China; 5 NHC Key Laboratory of study on abnormal gametes and reproductive tract, Anhui Medical University, Hefei, 230032 China; 6 Tsinghua-Peking Center for Life Sciences, Beijing, 100084 China

**Dear Editor,**

The first mitotic division in zygotes is crucial for the beginning of the life cycle for the human. After fertilization, zygotes reactivate cell cycle, both paternal and maternal genomes replicate and reprogram to become totipotent. In the meantime, the male and female pronucleus move to the center of the zygote and merge. Then zygotes enter the metaphase, and sister chromatids separate into two daughter cells (Eckersley-Maslin et al., 2018; Reichmann et al., 2018). This is a sensitive time window and many perturbances may cause the first mitosis to fail.

We discovered a family that the female adults were infertile. One female patient (proband III-7) underwent *in vitro* fertilization (IVF) treatment. The proband had regular menstrual cycles and sex hormone levels. She had undergone only one IVF cycle with classical controlled ovarian hyperstimulation protocol. Seventeen oocytes were retrieved and fertilized *in vitro*. Eight out of 15 metaphase II (MII) oocytes presented two obvious pronuclei during the fertilization checking. However, all embryos failed to complete the first cleavage (Table 1). Further investigation found that one sister (III-4) had undergone three failed IVF attempts for the same reason, two other sisters (III-3 and III-6) were infertile for unknown causes, and two sisters (III-2 and III-5) had healthy children. With the patients’ consent, whole-exome sequencing was carried out in all of the infertile subjects. After the pedigree analysis, we found that a heterozygous missense variant in the checkpoint kinase 1 gene (*CHEK1*), c.1325G>A (p. Arg442Gln), namely R442Q, was shared by all the infertile sisters (Fig. 1A). The variant was confirmed by Sanger sequencing in the whole family members (Fig. 1B). The results suggested that each infertile subject inherited the variant from their father II-8, and the variant was segregated within the whole family (Fig. 1A and 1B). CHEK1 protein plays an essential role in the DNA damage response (Sidi et al., 2008; Reinhardt and Yaffe, 2009) and is conserved across species (Fig. S1A). Interestingly, although the same *CHEK1* R442Q variant is expressed in somatic cells, based on RT-PCR and Sanger sequencing analysis of the peripheral blood samples from III-2, 3, 4, 5, 6 and 7 (Figs. 1C, S1B and S1C), heterozygous carriers are all healthy. We also analyzed the transcriptome profile of human oocytes, 1 and 2-cell embryos, *CHEK1* transcripts are present at high levels in germinal vesicle (GV) and MII oocytes, zygotes, and 2-cell embryos (Fig. 1D). Therefore, it should exist as a maternal protein.

**Table 1:** Oocyte and embryo phenotypes of IVF attempt for proband III-7 female patient carrying the *CHEK1* c.1325G>A (R442Q) variant.

Individual	Age (years)	Duration of infertility (Years)	IVF and ICSI attempts	Retrieved	MII	0PN	2PN	Cleaved fertilized oocytes
				oocytes	oocytes	oocytes	oocytes	
III-7	29	2	First IVF	17	15	7	8	0

**Figure 1:**
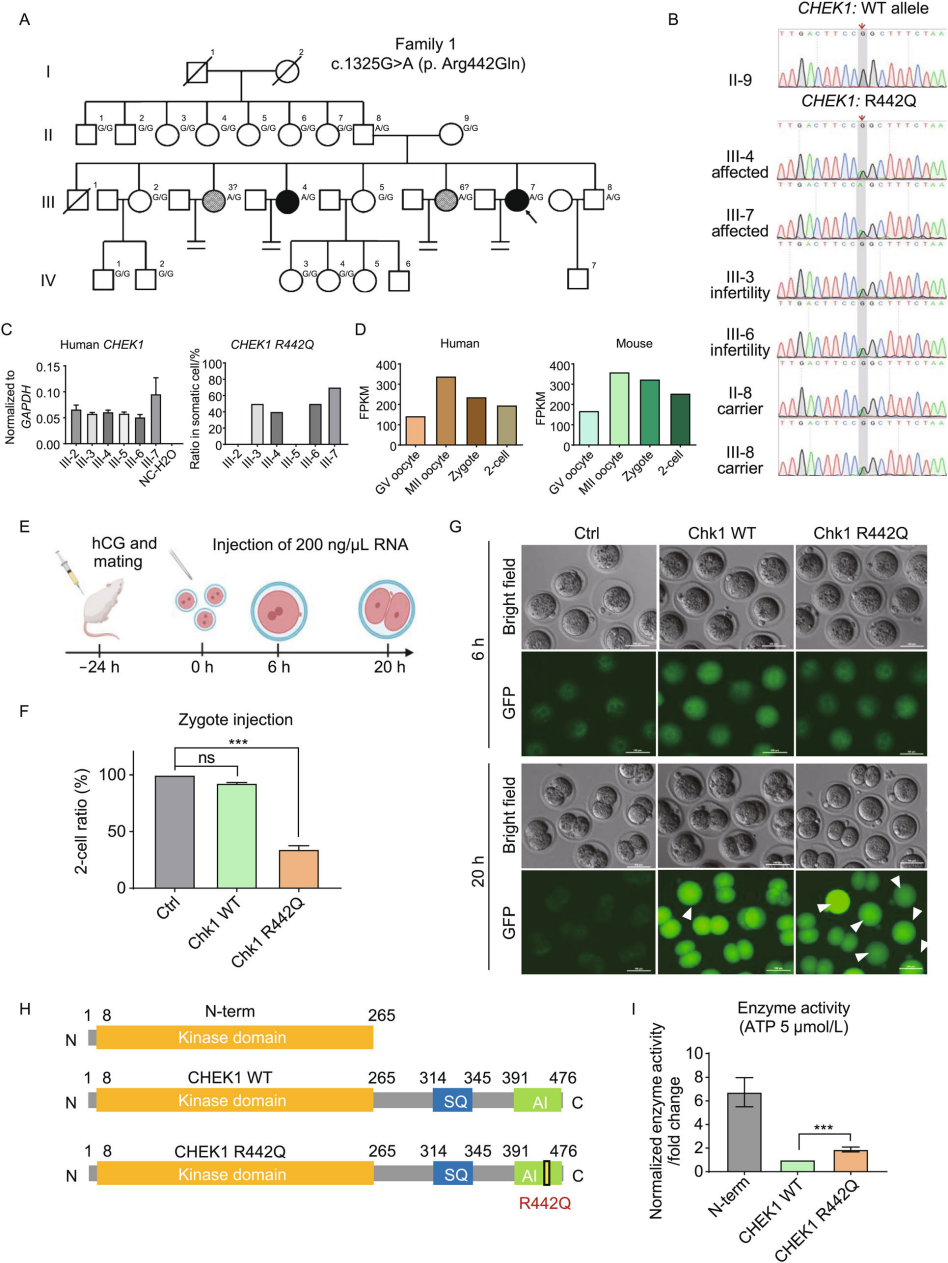
**First mitosis failure in zygotes from** ***CHEK1*** **c.1325G>A (p. Arg442Gln) females.** (A) Genetic analysis in the families carrying *CHEK1* c.1325G>A (p. Arg442Gln). Black circles indicate the affected individuals, and the circles with slashes indicate the suspected individuals (III-4 and III-7 had been confirmed during IVF cycles, III-3 and III-6 females were infertile, possibly caused by the arrest of the embryo cleavage). The black arrowhead indicates the proband. (B) Sanger sequencing result of *CHEK1* c.1325G>A (R442Q) mutation in affected, infertile, and carrier individuals. Double peak at the 1,325 position highlights the mutated nucleotide. (C) QPCR result confirmed that *CHEK1* R442Q transcripts are present in patients’ somatic cells. Left panel, the expression levels of CHEK1 mRNA in human blood cells. Right panel, the ratio of *CHEK1* R442Q mutant transcript in patient somatic cells verified by Sanger sequencing. (D) *CHEK1* expression levels in human and mouse GV oocyte to 2-cell embryo based on RNA-Sequencing data from GSE101571, GSE36552, and GSE71434. (E) Schematic of overexpressing *Chk1* WT and R442Q in mouse zygotes by mRNA microinjection (Created with BioRender.com). (F) Bar plot showing Chk1 R442Q arrest the first cleavage. Ctrl, *n* = 23. WT, *n* = 48. R442Q, *n* = 51. All pooled from 2 separate experiments; one-way analysis of variance (ANOVA), Bonferroni’s test for individual comparisons. Bars are means ± s.e.m. (G) Representative live cell images of zygotes microinjected with mRNA encoding Chk1 WT and R442Q GFP fusion protein. White arrowheads point to the arrested embryos. Time is as indicated. Scale bar, 100 μm. (H) Schematic of human CHEK1 protein domains. (I) CHEK1 R442Q protein showed higher kinase enzyme activity compared to WT. Luciferase based kinase assay normalized to WT group. 9 tests from 3 separate experiments. Two-sided Student’s *t*-test. Columns are means ± s.e.m

To test how CHEK1 R442Q protein affects zygote division, we cloned mouse Chk1, whose amino acid sequence is 93.07% identical to that of the human CHEK1, and generated the same Chk1 R442Q mutation as found in the infertile patient. Upon overexpression of wild type (WT) or R442Q Chk1 protein in mouse zygotes by mRNA injection, we found that more than 60% of the Chk1 R442Q zygotes failed to divide into 2-cell embryos, while overexpression of Chk1 WT did not affect the first mitosis (Fig. 1E–G). It has been shown that overexpression of Chk1 in mouse GV oocytes caused meiosis I arrest (Chen et al., 2012). Interestingly, when we overexpressed Chk1 WT and R442Q in GV oocytes, the R442Q caused significantly more oocyte arrest in metaphase I than WT (Fig. S2C–E). We analyzed Chk1 protein localization based on the immunofluorescence signal, the Chk1 WT and R442Q proteins localized both in nucleus and cytoplasm, with a stronger localization at nucleus and on the spindle during mitosis and meiosis (Fig. S2A and S2F). The amount of overexpressed mutant protein is less than the overexpressed WT protein, based on their respective mean fluorescence intensity (MFI) (Fig. S2B and S2G), suggesting that the R442Q change did not stabilize Chk1 protein.

As the activation of CHEK1 kinase in response to DNA damage will cause cell cycle arrest in the G_2_ phase (Reinhardt and Yaffe, 2009), we hypothesize that the R442Q mutation may cause an increase in CHEK1 protein kinase activity. Therefore, we generated the N-terminus kinase domain, the full-length, and the full-length R442Q mutant CHEK1 proteins and performed the kinase assay (Fig. 1H). Indeed, the N-terminus kinase domain had very high kinase activity, as reported by several studies (Goto et al., 2015; Han et al., 2016; Emptage et al., 2017). The R442Q mutant protein had about twice as high kinase activity as the WT protein (Figs. 1I, S1D and S1E).

There have been many studies about *CHEK1* and checkpoint kinase 2 (*CHEK2*) function in the context of human cancers. We looked for identified disease-related variants within *CHEK1* and *CHEK2* genes in the Human gene mutation database (HGMD). There are only 4 entries for *CHEK1* but 345 entries for *CHEK2* (Table S1). Four variants in *CHEK1* are all within the sequence encoding the kinase domain and associated with cancers. A high proportion of *CHEK2* variants are related to breast cancers. There was no report about the *CHEK1* c.G1325A, p.R442Q mutation. We speculate that this is likely due to female infertility caused by this mutation.

We next ask whether Chk1 R442Q may induce more DNA damage in zygotes. Interestingly, overexpression of Chk1 R442Q reduced γH2AX staining in the male pronucleus (Figs. 2A, 2B and S3A). Since R442Q mutant protein has higher kinase activity than the WT protein, we used a CHEK1 inhibitor CCT244747 to treat zygotes overexpressing WT or R442Q. As expected, CCT244747 rescued the first mitosis arrest caused by R442Q, nearly all treated R442Q zygotes divided into 2-cell embryos (Fig. S3B). However, regular CCT244747 dosage also increased DNA damage, as shown by stronger γH2AX staining in treated WT or R442Q embryos (Fig. S3C). Since R442Q mutant has approximately double kinase activity than WT protein, we reasoned that reducing the kinase activity close to the normal level might be sufficient to overcome the first mitotic arrest. Indeed, 30 nmol/L of CCT244747 permitted R442Q zygotes to divide into 2-cell embryos and did not increase the γH2AX signal (Figs. 2C, 2D and S3D–F). Moreover, 60% of 30 nmol/L CCT244747 treated R442Q zygotes developed to the blastocyst stage (Fig. 2E–H). We examined the expression of pluripotency genes and found that Oct4 and Nanog proteins are highly expressed in the inner cell mass of CCT244747 treated R442Q blastocysts (Fig. 2I). Treated WT and R442Q blastocysts also had a similar number of total cells and Oct4 and Nanog positive cells (Fig. 2J and 2K), suggesting that their development was not compromised.

**Figure 2:**
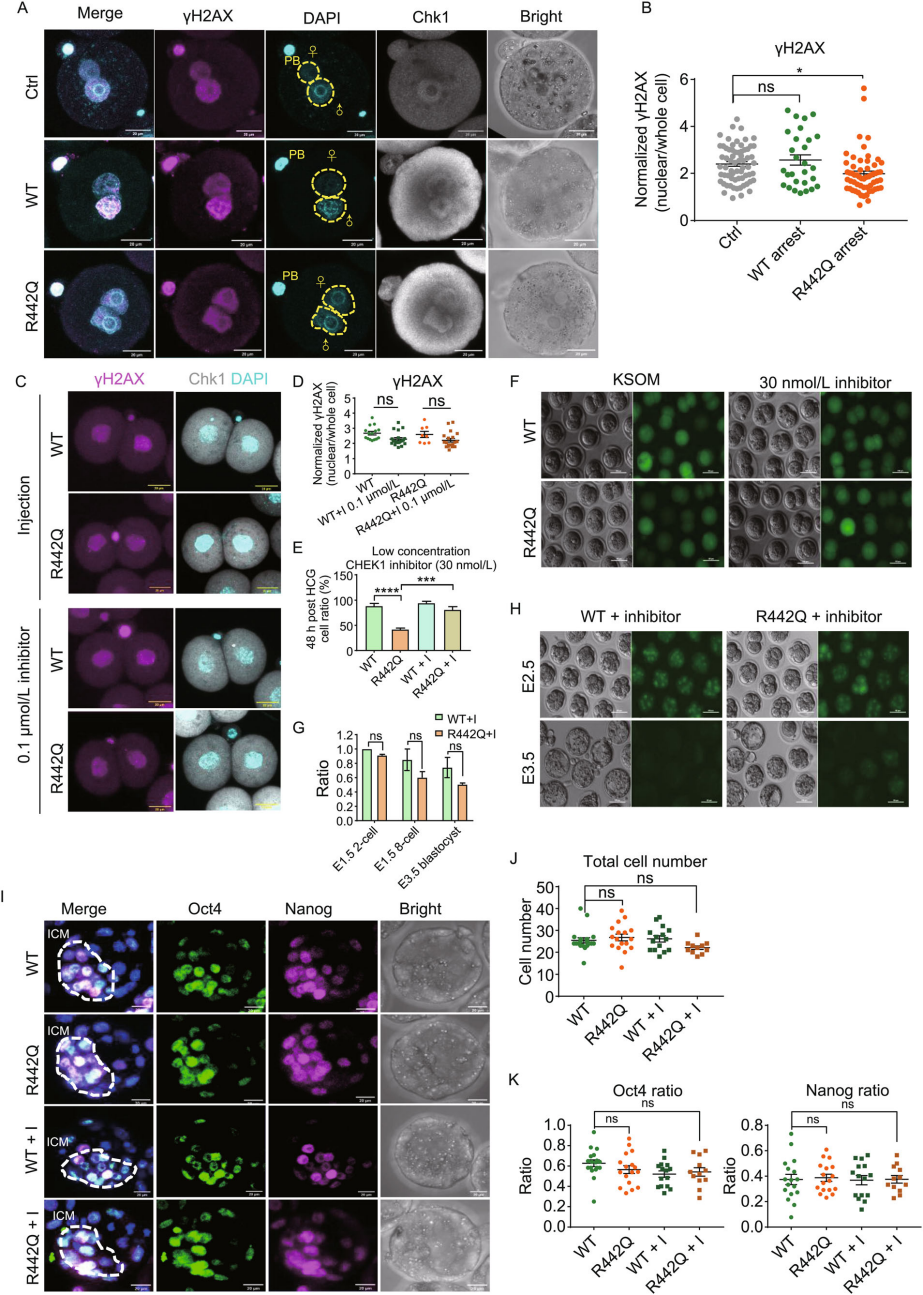
**A graded Chk1 activity differentially modulates DNA damage response and cell cycle progression in the zygote.** (A) Representative γH2AX immunostaining images of Ctrl, Chk1 WT, and R442Q zygotes. Yellow circles mark the paternal and maternal pronucleus. PB, polar body. Cyan, DAPI; pink, γH2AX; gray, Chk1. Scale bar, 20 μm. (B) Dot plot quantification of γH2AX signal in arrested R442Q mouse zygotes compared to control non-injected (Ctrl) and WT injected zygotes. Ctrl, *n* = 66, WT, *n* = 28. R442Q, *n* = 58. All pooled from 2 separate experiments. One-way analysis of variance (ANOVA), Bonferroni’s test for individual comparisons. Bars are means ± s.e.m. (C) γH2AX staining in CHEK1 inhibitor (0.1 μmol/L) rescued 2-cell embryos. Cyan, DAPI; pink, γH2AX; gray, Chk1. Scale bar, 20 μm. (D) Quantification of γH2AX signal in 0.1 μmol/L CHEK1 inhibitor-treated or non-treated WT and R442Q 2-cell embryos. Chk1 WT embryos with or without inhibitor treatment, *n* = 15 and 21. Chk1 R442Q embryos with or without inhibitor treatment, *n* = 8 and 20. One-way analysis of variance (ANOVA), Bonferroni’s test for individual comparisons. Bars are means ± s.e.m. (E) 30 nmol/L CHEK1 inhibitor treatment rescued the development of Chk1 R442Q zygotes. Chk1 WT embryos with or without 30 nmol/L inhibitor treatment, *n* = 98 and 106. Chk1 R442Q embryos with or without 30 nmol/L inhibitor treatment, *n* = 106 and 101. All pooled from 4 separated experiments. One-way analysis of variance (ANOVA), Bonferroni’s test for individual comparisons. Bars are means ± s.e.m. (F) Representative images of Chk1 WT and R442Q injected embryos with or without 30 nmol/L inhibitor treatment at 48 h post HCG. Scale bar, 100 μm. (G) 30 nmol/L CHEK1 inhibitor-treated embryos can develop to the blastocyst stage. Chk1 WT, *n* = 48. Chk1 R442Q, *n* = 46. All pooled from 2 separate experiments. Two-way analysis of variance (ANOVA), Bonferroni’s test for individual comparisons. Bars are means ± s.e.m. (H) Representative images of 8-cell and blastocysts developed from zygotes injected with mRNA of Chk1 WT and R442Q then treated with 30 nmol/L CHEK1 inhibitor. The stage is as indicated. Scale bar, 100 μm. (I) Immunostaining of Oct4 and Nanog in blastocysts developed from WT or R442Q zygotes treated with 30 nmol/L CHEK1 inhibitor. The dashed line highlights the ICM region. Blue, DAPI; green, Oct4; pink, Nanog. Scale bar, 20 μm. ICM, inner cell mass. (J) Statistic analysis of total cell number in blastocysts derived from Chk1 WT and R442Q injected zygotes with or without inhibitor treatment. WT embryos with or without 30 nmol/L inhibitor treatment, *n* = 19 and 15. R442Q embryos with or without 30 nmol/L inhibitor treatment, *n* = 17 and 13. All pooled from 2 separate experiments. One-way analysis of variance (ANOVA), Bonferroni’s test for individual comparisons. Bars are means ± s.e.m. (K) Statistic analysis of the ratio of Oct4, and Nanog positive cells, related to (J)

We next performed transcriptome profiling of zygotes, 2-cell embryos, and blastocysts injected with mRNA encoding WT or R442Q with or without low dosage CHEK1 inhibitor treatment (Fig. S4A). Principal component analysis (PCA) and heatmap clustering showed that 1-cell arrested R442Q zygotes clustered together with PN5 WT zygotes (Fig. S4B and S4C), suggesting that the minor zygotic genome activation was not affected. At the late 2-cell stage, the transcriptome of R442Q embryos, which managed to divide, clustered together with WT 2-cell embryos (Fig. S4B and S4C), indicating that the major zygotic genome activation occurred in these embryos. The CHEK1 inhibitor rescued WT and R442Q blastocysts clustered together, consistent with our previous observations (Figs. S4B, S4C and 2I–K). We analyzed differentially expressed transcripts in WT and R442Q zygotes, 2-cell embryos, and CHEK1 inhibitor rescued WT and R442Q blastocysts. The expression of zygotic genome activation genes and key developmental genes were largely unaffected (Fig. S4D and Table S2). Finally, we transferred CHEK1 inhibitor rescued R442Q 2-cell embryos into female mice and found that a similar number of pups were born compared with the WT embryo transferred group (Fig. S4E–G). These pups grew up to adulthood and had similar number of offspring as WT pups treated with or without the CHEK1 inhibitor (Fig. S4H). These results suggest that the low concentration of CHEK1 inhibitor treatment may relieve zygotes from the first mitotic division arrest caused by a slight tightening up of DNA damage checkpoint, and the rescued embryos would develop normally.

The discovery of the *CHEK1* R442Q mutation and the first zygotic mitosis arrest phenotype in human reproduction has important implications. All the heterozygous female carriers are infertile, presumably due to failed mitosis in zygotes. As the zygotes contained mostly maternal proteins inherited from the egg, both CHEK1 WT and R442Q proteins should be present in the carrier’s eggs. Based on the clinical observation during the IVF treatment, CHEK1 R442Q did not affect oocyte meiosis, suggesting that a small elevation in CHEK1 kinase activity does not block the first meiotic division in women. The II-8 and III-8 male carriers are healthy and do not have fertility problems, indicating that *CHEK1* R442Q does not affect sperm meiosis in men. CHEK1 is a ubiquitously expressed protein, and all the heterozygous individuals are healthy without cancer or autoimmune diseases based on informed consent health history surveys. Thus, CHEK1 R442Q should not affect most mitotic division of embryonic or adult cells. All the heterozygous females inherited the *CHEK1* R442Q mutation from their father as their mother has two copies of the normal *CHEK1* gene. Since the paternal genome starts to express after the major zygotic genome activation during the 4 to 8-cell stage in humans (Eckersley-Maslin et al., 2018), the CHEK1 R442Q protein derived from the mutated paternal copy of *CHEK1* will likely be present from the 8-cell stage onwards. Therefore, the small elevation of CHEK1 kinase activity does not appear to arrest cell division after the 8-cell stage in human embryos. Thus, the CHEK1 R442Q protein seemed to affect the first mitotic division most seriously, indicating that the first mitosis is particularly sensitive to DNA damage checkpoint activation in humans. Moreover, the DNA damage checkpoint threshold may depend on the cell type, developmental stage, and species, based on the human phenotype and our results obtained in the mouse system. When we treat Chk1 R442Q overexpressing mouse zygotes with a low dosage of CHEK1 inhibitor, they can escape the first mitotic arrest and develop normally. CHEK1 inhibitors are often used as cancer drugs. They inhibit the DNA damage checkpoint, making cancer cells accumulate DNA damage and die (Sidi et al., 2008; Reinhardt and Yaffe, 2009). Surprisingly, although a high dosage of CHEK1 inhibitor also induced DNA damage in zygotes, a low dosage of CHEK1 inhibitor rescued R442Q overexpression induced first mitosis arrest without eliciting any DNA damage judging by the γH2AX staining, and the embryos developed normally. Chk1 R442Q overexpression also did not induce γH2AX signal. The above results suggested that small up or down-regulation of CHEK1 kinase activity may not affect genome stability. Our findings also imply the possibility to use low dosage CHEK1 inhibitor treatment to release cells from the low-level genome stress-induced cell cycle block during zygote mitosis or other sensitive periods.

In summary, our results revealed an unexpected zygote mitotic checkpoint, which is extremely sensitive to the CHEK1 kinase activity. The fine-tuning of the DNA damage checkpoint permits the arrested one-cell embryos to overcome the first mitotic block and develop into healthy animals. These findings have important implications in assisted human reproduction and genetic counseling in women with zygotic division failure.

## Supplementary Information

Below is the link to the electronic supplementary material.

Supplementary material 1 (PDF 10678 kb)
