# Supplementary material for: Maternal heterozygous mutation in CHEK1 leads to mitotic arrest in human zygotes
Source: Protein Cell. 2021 May 4;13(2):148–54. doi: 10.1007/s13238-021-00844-9 (PMC8783945; doi:10.1007/s13238-021-00844-9)
Supplement: Supplementary file 1 — Supplementary material 1 (PDF 10678 kb) [file 13238_2021_844_MOESM1_ESM.pdf]

Figure S1: *CHEK1* gene locus and *CHEK1* c.1325G>A (p. Arg442Gln) expression in somatic cells of carriers.

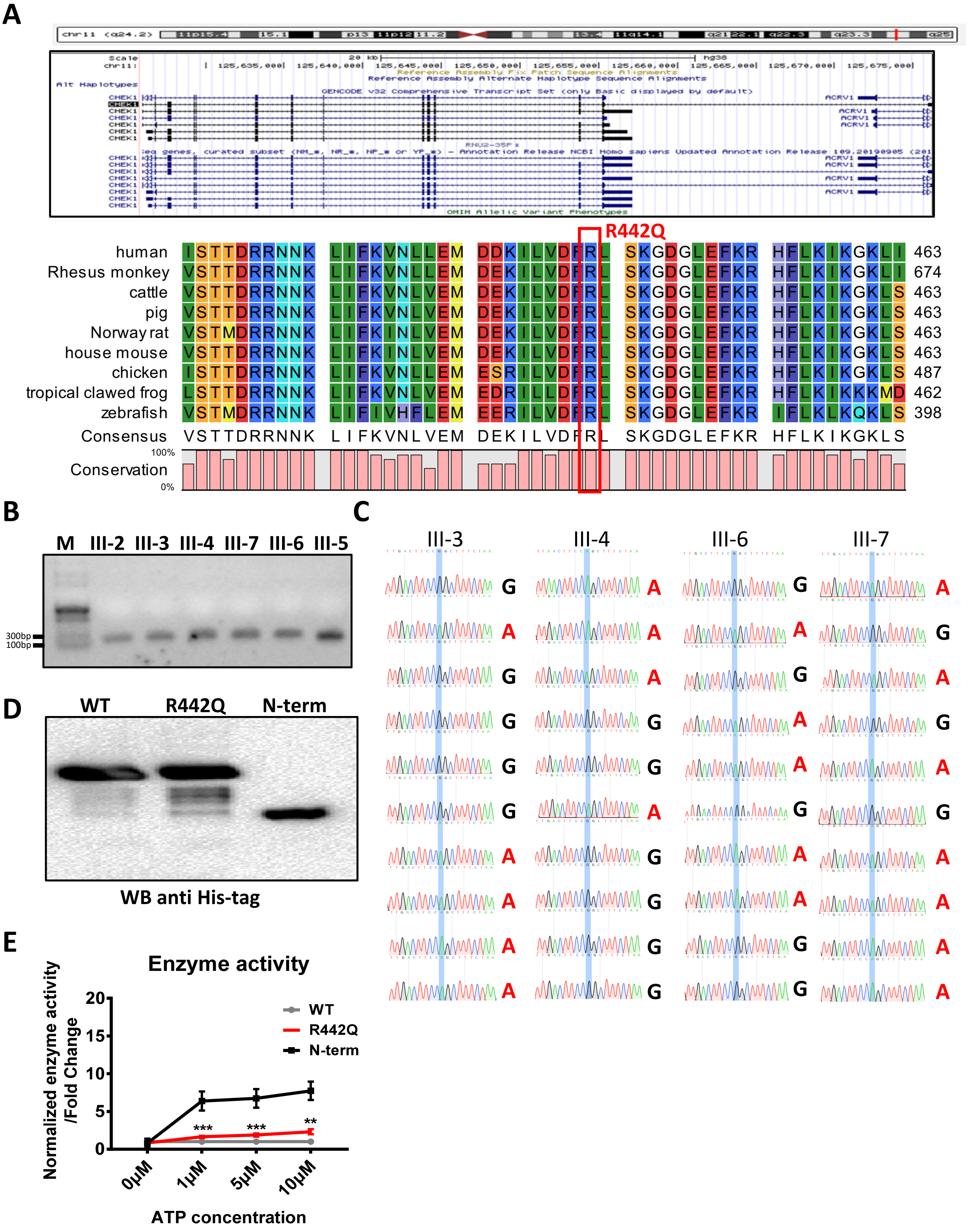

Figure S2: Chk1 localization in mouse preimplantation embryos and oocytes.

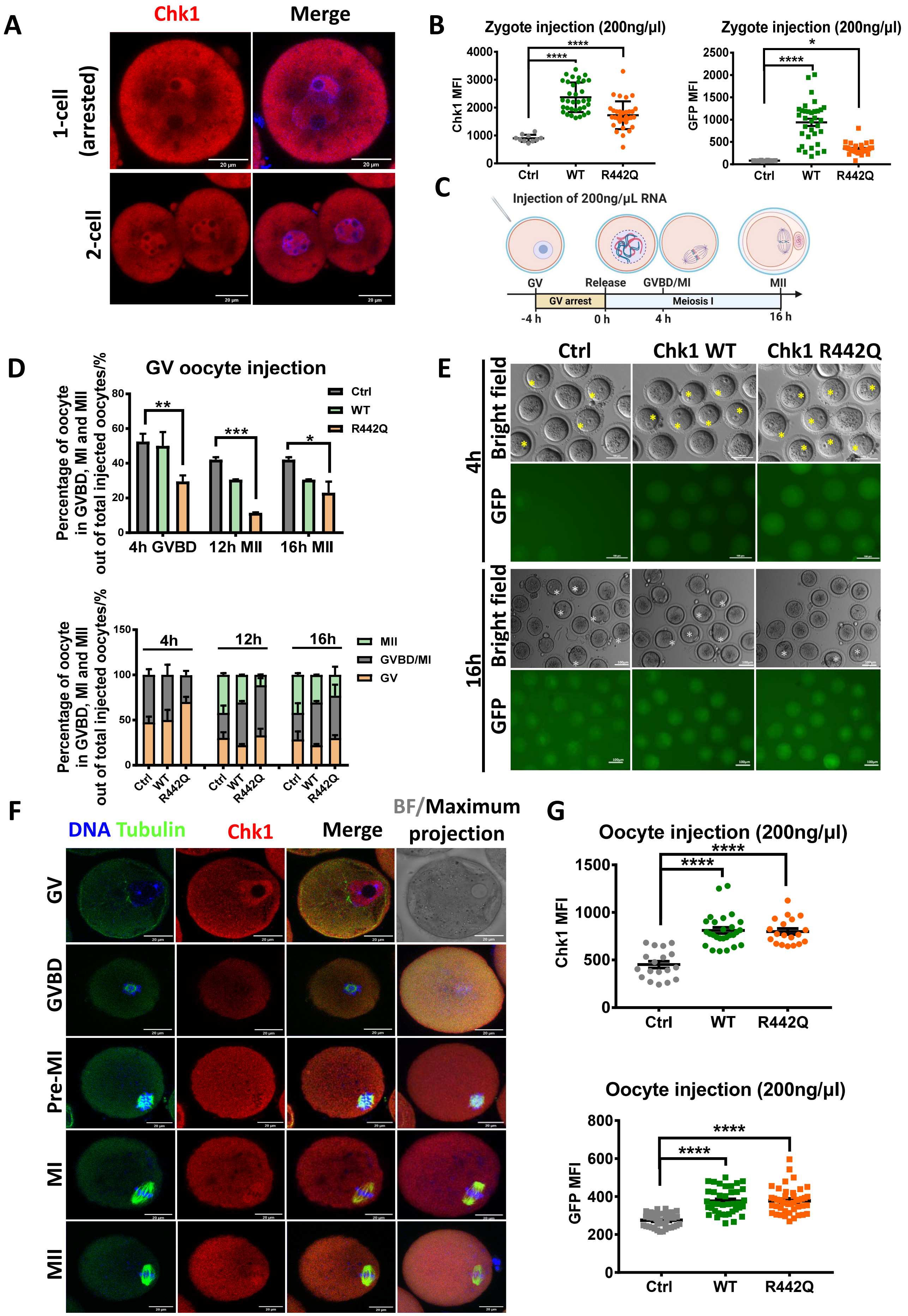

Figure S3: A high concentration of CHEK1 inhibitor treatment caused DNA damage.

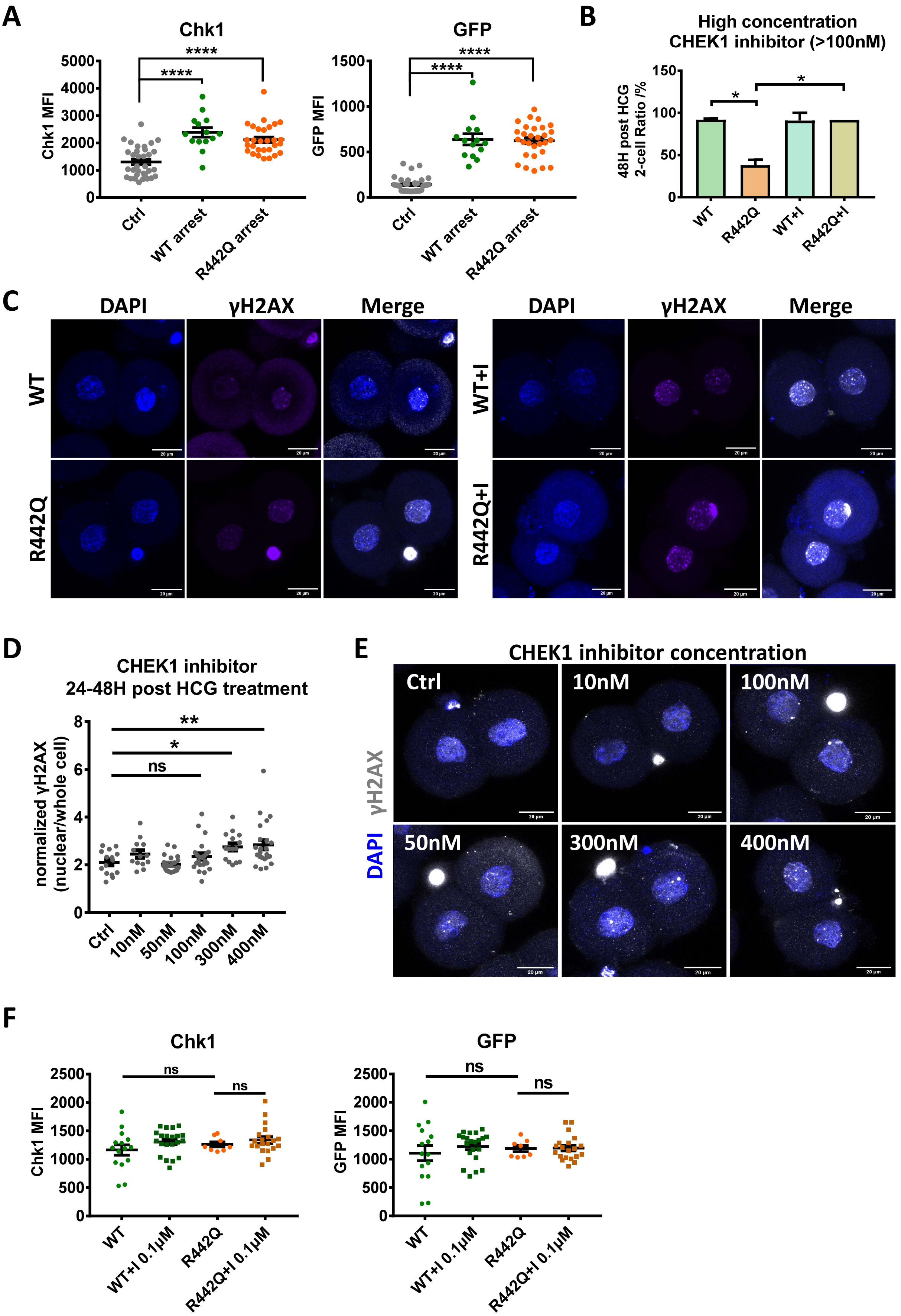

Figure S4: A low dosage of CHEK1 inhibitor treatment does not affect zygotic genome activation and permitted normal embryo development.

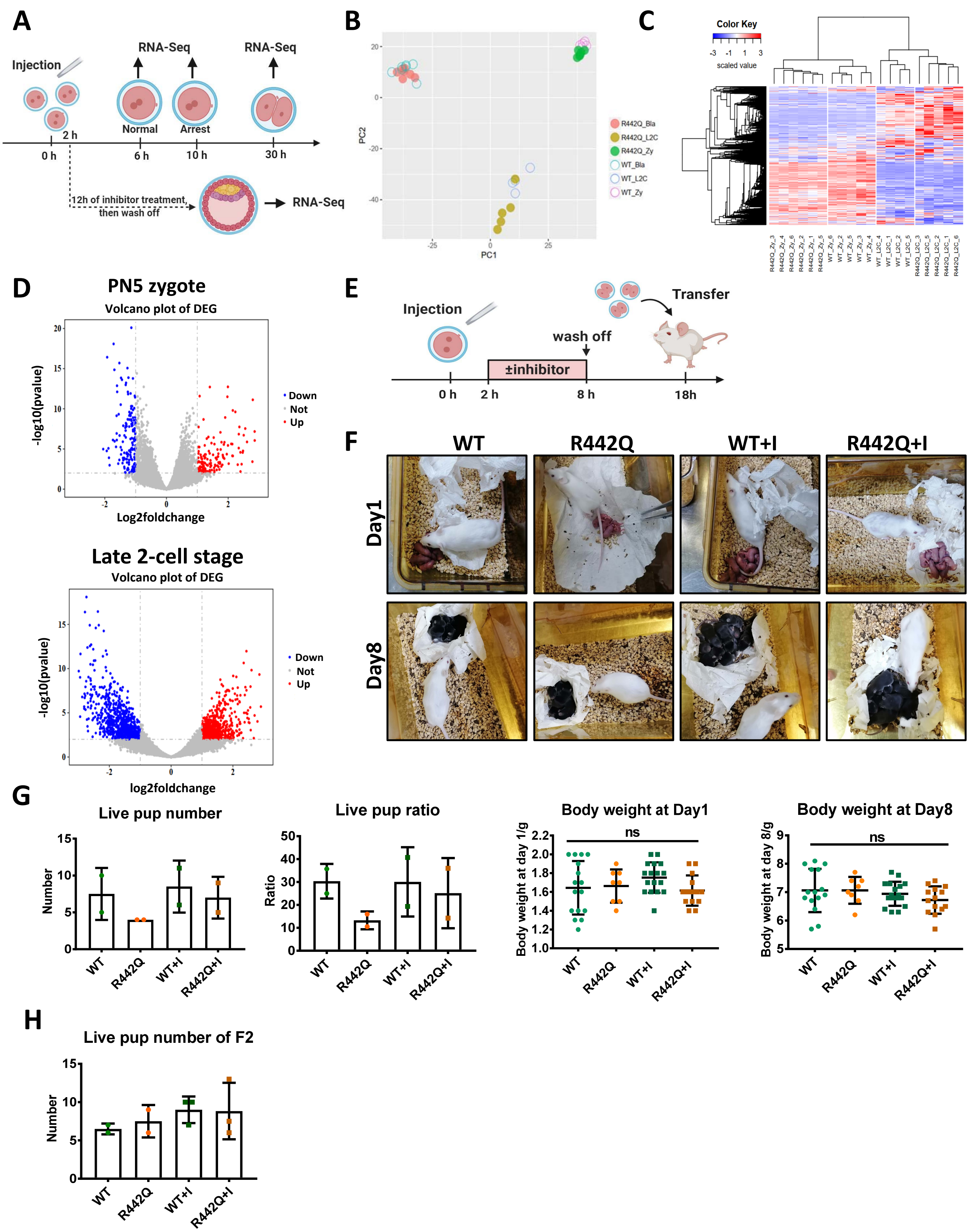

Supplemental Figure Legends

Figure S1: *CHEK1* gene locus and *CHEK1* c.1325G>A (p. Arg442Gln) expression in somatic cells of carriers.

(A) Human *CHEK1* gene locus and amino acid sequence of CHEK1 protein C-terminus. The Arg442 in human CHEK1 protein is conserved among all species.

(B) *CHEK1* is expressed in somatic cells of carriers verified by RT-PCR and agarose gel electrophoresis.

(C) Detection of the ratio of *CHEK1* R442Q mutant RNA transcript by Sanger sequencing. 10 colonies were picked and sequenced for each sample.

(D) Western blot showing His-tag purified CHEK1 N-term, WT, and R442Q proteins.

(E) Kinase assay showed CHEK1 R442Q mutant protein had increased kinase activity compared to WT protein. n = 9 from 3 independent experiments. Two-sided Student's t-test. Columns are means ± s.e.m.

Figure S2: Chk1 localization in mouse preimplantation embryos and oocytes.

(A) Chk1 protein localizes to both nuclear and cytoplasm in mouse early embryos. Red, Chk1; blue, DAPI. Scale bar, 20 μm.

(B) Microinjection of mRNA encoding Chk1 WT and R442Q GFP fusion protein leads to an elevation of Chk1 and GFP protein expression in zygotes and 2-cell embryos. Ctrl, n=10. WT, n=34. R442Q, n=33. All pooled from 2 separate experiments. One-way analysis of variance (ANOVA), Bonferroni’s test for individual comparisons. Bars are means ± s.e.m.

(C) Schematics of overexpressing Chk1 WT and R442Q in mouse oocytes (Created with BioRender.com). Microinjected GV oocytes were first blocked in the GV stage with Cilostamide for 4h. GVBD rate and MII rate 4h, 12h, 16h after release from blocking were counted. GV, germinal vesicle; GVBD, germinal vesicle break down; MI, metaphase I; MII, metaphase II.

(D) Bar plot showing Chk1 R442Q delayed mouse oocyte development. Ctrl, n=50. WT, n=59. R442Q, n=52. All pooled from 2 separate experiments. Bars are means ± s.e.m. Upper panel, statistical analysis of the percentage of GVBD 4h after release, MII 12h, and 16h after release. Two-way analysis of variance (ANOVA), Bonferroni’s test for individual comparisons. Lower panel, composite bar graph of percentages of GV, GVBD, and MII oocytes of Ctrl, Chk1 WT,

and R442Q groups. Y-axis, Percentage of oocyte in GVBD, MI and MII out of total injected oocytes.

(E) Representative live cell images of oocytes microinjected with mRNA encoding Chk1 WT and R442Q GFP fusion proteins. Yellow and white stars point out GV arrested oocytes (4h), and oocytes progressed to the MII stage (16h). Scale bar, 100 μm.

(F) Chk1 protein locates in the germinal vesicle and on the spindle of mouse oocytes. Red, Chk1; blue, DAPI; green, Tubulin. Scale bar, 20 μm.

(G) Microinjection of mRNA encoding Chk1 WT and R442Q GFP fusion protein into GV oocytes leads to an elevation of Chk1 and GFP protein expression. Ctrl, n=38. WT, n=39. R442Q, n=44. All pooled from 2 independent experiments. One-way analysis of variance (ANOVA), Bonferroni's test for individual comparisons. Bars are means ± s.e.m.

Figure S3: A high concentration of CHEK1 inhibitor treatment caused DNA damage.

(A) Chk1 and GFP proteins were expressed at similar levels in zygotes injected with mRNA encoding Chk1 WT and R442Q GFP fusion proteins. Related to Fig. 2A. n=66 Ctrl zygotes, n=28 WT oocytes, and n=58 R442Q oocytes, pooled from 2 separate experiments. One-way analysis of variance (ANOVA), Bonferroni's test for individual comparisons. Bars are means ± s.e.m.

(B) Higher concentrations of CHEK1 inhibitor (>100 nM) rescued Chk1 R442Q embryos to the 2-cell stage at 48 h post HCG. Chk1 WT injected embryos with or without inhibitor treatment, n=41 and 29; Chk1 R442Q injected embryos with or without inhibitor treatment, n=44 and 24. All pooled from 2 separate experiments. One-way analysis of variance (ANOVA), Bonferroni's test for individual comparisons. Bars are means ± s.e.m.

(C) gH2AX staining in 500 nM CHEK1 inhibitor treated WT and R442Q 2-cell embryos. Blue, DAPI; purple, γH2AX. Scale bar, 20 μm.

(D) CHEK1 inhibitor concentration lower than 100 nM did not increase γH2AX signal in mouse early embryos. n=15,13,21,22,15 and 23 for Ctrl, 10 nM, 50 nM, 100 nM, 300 nM and 400 nM inhibitor-treated groups. One-way analysis of variance (ANOVA), Bonferroni's test for individual comparisons. Bars are means ± s.e.m.

(E) gH2AX staining of mouse embryos treated with different concentrations of CHEK1 inhibitor 48 h post HCG from (D). Blue, DAPI; gray, γH2AX. Scale bar, 20 μm.

(F) Comparison of Chk1 and GFP protein levels in 2-cell embryos injected with mRNA encoding Chk1 WT and R442Q GFP fusion protein at PN3 zygote stage and treated with or without 0.1 μM of CHEK1 inhibitor, related to Fig. 2C. WT embryos with or without inhibitor treatment, n=15 and 21. R442Q embryos with or without inhibitor treatment, n=8 and 20. All pooled from 2 separate experiments. One-way analysis of variance (ANOVA), Bonferroni's test for individual comparisons. Bars are means ± s.e.m.

Figure S4: A low dosage of CHEK1 inhibitor treatment does not affect zygotic genome activation and permitted normal embryo development.

(A) Schematic of Chk1 WT and R442Q embryos used for RNA-Seq analysis (Created with BioRender.com). Chk1 WT or R442Q mRNA were injected into mouse zygotes. One-cell embryos were harvested at 6 h (WT) or 10 h (R442Q) post-injection. Two-cell embryos were collected 30 h post-injection. For blastocysts, Chk1 WT and R442Q injected zygotes were first treated with 30nM inhibitor overnight. After washing off the inhibitor, 2-cell embryos were cultured to the blastocyst stage and used for RNA-seq.

(B) PCA analysis of RNA-Seq data from single WT and R442Q embryos.

(C) Heatmap clustering of WT and R442Q zygotes and 2-cell stage embryos.

(D) Volcano plot depicting significantly differentially expressed genes in zygotes and 2-cell embryos injected with mRNA encoding Chk1 WT (n=5 zygotes, n=4 2-cell embryos) and R442Q (n=6 zygotes, n=5 2-cell embryos).

(E) Schematic of Chk1 WT and R442Q injected zygotes with or without 30 nM of CHEK1 inhibitor treatment, followed by transferring to pseudopregnant female mice (Created with BioRender.com).

(F) Chk1 WT and R442Q mouse embryos treated with 30 nM of inhibitor developed to live pups with after transplantation.

(G) Chk1 WT and R442Q embryos treated with or without 30 nM of inhibitor developed to live pups with normal body weight after transplantation. n=15, 8, 17, and 14 live pups of Chk1 WT, R442Q, WT with inhibitor, and R442Q with inhibitor group. All pooled from 2 separate experiments.

(H) mice from upper panel had normal fertility and produced F2 live pups. n=19, 15, 34, and 34 live pups of Chk1 WT, R442Q, WT with inhibitor, and R442Q with inhibitor group.

Supplemental Tables

Table S1. List of all published *CHEK1* and *CHEK2* variants, related diseases and publications.

Information source: the Human Gene Mutation Database (HGMD®, http://www.hgmd.cf.ac.uk/ac/index.php) and HGMD Professional (https://digitalinsights.qiagen.com/products-overview/clinical-insights-portfolio/human-gene-mutation-database/).

Table S2. Differentially expressed genes (DEGs) in mouse preimplantation embryos with Chk1 WT or R442Q overexpression.

Sheet names: R442Q Zy Dn, R442Q Zy Up, R442Q L2C Dn, R442Q L2C Up, showing list of genes downregulated (Dn) or upregulated (Up) in mouse zygotes (Zy) or late 2-cell embryos (L2C) developed from Chk1 R442Q zygotes, compared to Chk1 WT zygotes.

Sheet names: R442Q Bla Dn, R442Q Bla Up, showing list of genes downregulated or upregulated in mouse blastocysts developed from CHEK1 inhibitor treated Chk1 R442Q zygotes compared to treated Chk1 WT zygotes.

Supplemental Methods

Patient consent and ethical approvement

The proband III-7 and her family were recruited for this study from the First Affiliated Hospital of Anhui Medical University. The human sample collection and analysis was approved by the Ethics Committee for Clinical Medical Research of the First Affiliated Hospital of Anhui Medical University and carried out with informed consent.

Whole-exome sequencing (WES) analysis

Libraries were generated using the Agilent SureSelect Human All Exon V6 kit (Agilent Technologies, USA) following the manufacturer's recommendations. WES was carried out on an Illumina NovaSeq6000 sequencer with pair end 150 bp (PE150) for each reaction. The original sequencing reads were aligned to the reference genome GRCh37/hg19, variants including single nucleotide polymorphisms (SNPs) and short insertion and deletion (INDELs) were called using the GATK software program. The identified variants were further annotated using ANNOVAR software. The criteria used for filtering the desired variants were as follows (i) missense, nonsense, frameshift, or splice site variants; (ii) variants with minor allele frequency < 1%. The minor allele frequency data were obtained by referring to the following databases: Genome Aggregation Database (gnomAD, http://gnomad.broadinstitute.org/), 1000 Genomes (1000G, http://browser.1000genomes.org/index.html), and the NHLBI Exome Sequencing Project (ESP6500).

Quantitative PCR

Total RNA of patient and control blood samples were extracted with TRIZOL (Invitrogen). 1μg RNA of each sample was used for reverse transcription with Superscript II (Invitrogen). Q-PCR reactions were performed using GoTaq qPCR Master Mix (Promega) in a CFX96 Real-Time System (Bio-Rad). The relative expression level of each gene was normalized against the Ct (Critical Threshold) value of the house-keeping gene GAPDH using the Bio-Rad CFX Manager program. For detecting the mutation transcript in the patient's somatic cells, qPCR products were purified by agarose gel electrophoresis, ligated to pEASY-T5 Zero plasmid (TransGen CT501-01), and sequenced using the M13 forward primer. QPCR primer used in this study:

hCHEK1-F: CAACAACTGATAGGAGAAACAATAAACT;

hCHEK1-R: CAAATCTTCTGGCTGCTCACAATA;

hGAPDH-F: TGATGACATCAAGAAGGTGGTGAAG;

hGAPDH-R: TCCTTGGAGGCCATGTGGGCCAT.

Mouse embryo culture and microinjection

All animal experiments were conducted following the Guide for the Care and Use of Animals for Research Purposes. The protocol for mouse embryo isolation was approved by the Institutional Animal Care and Use Committee and Internal Review Board of Tsinghua University. Embryos were collected from wild type C57BL/6 females (Charles River). Zygotes for RNA injection were collected from mated female mice 26-28 h post-HCG, then cultured in KSOM medium (Merck Millipore MR-106-D) in 37.5℃ 5% CO_2 incubator. GV oocytes were collected from the ovary of C57BL/6 female in M2 medium (Sigma M7167) supplemented with 10 μM Cilostamide (Cayman 14455). Microinjection of mRNAs at a concentration of 200 ng/μl into the mouse GV oocytes or PN3 zygotes was performed on a Leica microscope micromanipulator. The microinjected embryos were treated with or without CHEK1 inhibitor CCT244747 (MedChemExpress, HY-18175/CS-5919) at different concentrations as indicated.

Plasmid cloning and RNA synthesis

Mouse *Chk1* cDNA was cloned into the RN3P vector for in vitro transcription of mRNA. mRNAs were generated using the T3 mMESSAGE mMACHINE Kit according to the manufactures instructions (Ambion).

Immunostaining and confocal microscopy

For immunostaining, mouse oocytes or embryos were fixed in 1% PFA overnight, then permeabilized with 0.25% Triton X-100 for 20 min. Embryos were then blocked with 5% FCS at room temperature for 2 h and incubated with primary antibody overnight at 4℃， followed by washing in PBST and incubating with secondary antibody for 1 h at room temperature. Antibodies

used in this study are: CHEK1 (Abcam, ab32531), Oct4 (Abcam, ab19857), gamma H2A.X (phospho S139, Abcam, ab26350), Tubulin (Abcam, ab6160), Nanog (CST, 4903P). Secondary antibodies used are: Dylight 488-conjugated Goat Anti-Mouse (Thermo, 35502), Dylight 488-Goat Anti-Rabbit (Thermo, A32727), Dylight 549-conjugated Goat Anti-Mouse (Thermo, 35552), and Dylight 549-conjugated Goat Anti-Rabbit (Thermo, 35557). DNA was stained by DAPI (Sigma-Aldrich, 28718-90-3). Immunostaining images were acquired with a Nikon A1 confocal microscope using an oil-immersion 40× objective. Raw data were processed using open-source image analysis software Fiji ImageJ.

CHEK1 protein production and kinase assay

For human CHEK1 protein purification and kinase assay, N-term (1-289aa), WT full-length, and R442Q mutant full-length human *CHEK1* cDNAs were cloned from 293FT cells, fused with 6×His tag at the N-terminus, and cloned into Bac-to-Bac Baculovirus Expression System following the instructions of the manufactures (Invitrogen 10359-016).

For kinase assay, Sf9 insect cells cultured in suspension in ESF 921 Insect Cell Culture Medium (Expression, 96-001) were used for protein overexpression and purification. Baculovirus of His-tagged N-term (1-289aa), WT full length, and mutant full-length human *CHEK1* were introduced into the sf9 insect cells by Cellfectin II Reagent (Thermo, 10362100) following the instructions of the manufactures. Recombinant Proteins were purified with affinity Ni-NTA agarose from approximately 2×10^8 infected cells. Purified proteins were verified by Coomassie bright blue stain and Western blot with the antibody of CHEK1 and His-tag. The secondary antibodies used were HRP-conjugated Goat Anti-Rabbit (Jackson ImmunoResearch, 111-065-003), HRP-conjugated Goat Anti-Mouse (Jackson ImmunoResearch, 111-035-003). CHEK1 kinase assay was conducted using the kinase assay kit (Beyotime, S0150) with the substrate of Cdc25C-derived Chktide (Sigma-Aldrich, 12-373).

Database search for disease-related Human *CHEK1* and *CHEK2* variants

All published *CHEK1* and *CHEK2* variants were obtained by searching the Human Gene Mutation Database (HGMD®, http://www.hgmd.cf.ac.uk/ac/index.php) and HGMD Professional (https://digitalinsights.qiagen.com/products-overview/clinical-insights-portfolio/human-gene-mutation-database/). HGMD has adopted a policy of sub-categorising polymorphism entries. Polymorphisms may be allocated to the following possible categories:
DM (Disease causing mutation), pathological mutation reported to be disease causing in the report entered into HGMD. **DM?** (likely disease-causing mutation), the mutation reported in the literature is likely to be pathogenic, but the authors still have doubts, or follow-up studies have questioned the pathogenicity of the mutation. **DP** (Disease-associated polymorphism), A polymorphism reported to be in significant association with disease ($p<0.05$) that is assumed to be functional (e.g. as a consequence of location, evolutionary conservation, replication studies etc), although there may as yet be no direct evidence (e.g. from an expression study) of function. **DFP** (Disease-associated polymorphism with additional supporting functional evidence), A polymorphism reported to be in significant association with disease ($p<0.05$) that has evidence of being of direct functional importance (e.g. as a consequence of altered expression, mRNA studies etc.). **FP** (In vitro/laboratory or in vivo functional polymorphism), A polymorphism reported to affect the structure, function or expression of the gene (or gene product), but with no disease association reported as yet.

Transcriptome Profiling

For gene expression study in mouse preimplantation embryos, we used the SMART-seq2 protocol to amplify single embryo RNA [21]. Embryos were first lysed in hypotonic lysis buffer (Amresco, M334), followed by reverse transcription and pre-amplification. After AMPure XP beads purification, the cDNA libraries were tagmented by Tn5 and were subject to Illumina Nextera library preparation. All libraries were sequenced on Illumina HiSeq X-10 according to the manufacturer's instruction.

RNA-seq data processing

Adapter sequences were trimmed using TrimGalore (version 0.4.4). Clean reads were mapped to the mouse genome (mm10) using Bowtie2 (version 2.3.5) software with the Refseq annotation. Gene expression values were represented as FPKM (Fragments Per Kilobase per Million)

calculated by RSEM (version 1.2.28). Differential expression genes (DEGs) were analyzed using the DESeq2 package (version 1.20.0). The top 3000 highly expressed genes were used for comparason. Genes that satisfied the threshold “fold change ≥ 2, $p < 0.01$” were considered significant DEGs. DEGs from different stages are listed in separate sheets in supplementary table 2. Principle component analyses (PCA) were performed using R's “prcomp” function and drawn by ggplot2 package (version 3.1.0). The heatmaps were produced by the heatmap2 function of the gplots package (version3.0.1.1) with the hierarchical clustering method. The enrichment of GO was analyzed using clusterProfiler package (version 3.8.1).

Statistical analysis

Data are presented as mean ± standard error of the mean (SEM). Statistical significance was determined by Student’s t-test (two-tail) for two groups; one-way or two-way Analysis of Variance (ANOVA) for multiple groups using Graphpad software. $P < 0.05$ was considered significant.

Data availability

Raw and processed RNA-seq data are available at the Gene Expression Omnibus GEO by accession number GSE163765. All other relevant data are available from the corresponding author upon request.
